# Precedence for the Role of Indole with Pathogens

**DOI:** 10.1128/mBio.01599-19

**Published:** 2019-07-30

**Authors:** Thomas K. Wood, Jintae Lee

**Affiliations:** a Department of Chemical Engineering, Pennsylvania State University, University Park, Pennsylvania, USA; b School of Chemical Engineering, Yeungnam University, Gyeongsan, Gyeongbuk, South Korea; Johns Hopkins Bloomberg School of Public Health

**Keywords:** environmental microbiology, gastrointestinal infection, indole

## LETTER

Continuing the trend of impressive results demonstrating the importance of indole as an interspecies (1, 2) and interkingdom (3) signal that reduces pathogenicity (1), Kumar and Sperandio recently reported that indole represses the expression of virulence genes of the gastrointestinal tract pathogens enterohemorrhagic Escherichia coli (EHEC) and Citrobacter rodentium (4). However, we wish to point out several omissions from their paper.

With regard to the mechanism reported by Kumar and Sperandio for sensing indole through the histidine kinase sensor CpxA of EHEC (4), it is important to note that this was discovered 14 years earlier for E. coli by Hirakawa et al. (5). These researchers found that indole sensing in commensal E. coli requires the BaeSR and CpxAR two-component systems. Unfortunately, this contribution was not cited by Kumar and Sperandio (4).

Additional important omissions by Kumar and Sperandio (4) include that it is already established that indole reduces EHEC virulence, in that we showed 12 years earlier that indole repels EHEC (negative chemotaxis), reduces EHEC biofilm formation (a virulence trait), reduces EHEC motility, and reduces EHEC attachment to HeLa cells (a virulence trait) (6). Indole has also been shown to attenuate the pathogenicity of Staphylococcus aureus (7). Furthermore, indole has been shown previously to act as a true signal for E. coli (8), and it has been argued by us that indole is likely hydroxylated by oxygenases to become an even more potent signal in the gastrointestinal tract (8). Since commensal E. coli produces so much indole in the gastrointestinal tract, we have speculated that indole is the likely archetype for human hormones (2). Unfortunately, these references were not cited.

Furthermore, Kumar and Sperandio also failed to indicate that indole has been shown to reduce the virulence of Pseudomonas aeruginosa, another gastrointestinal tract pathogen, by decreasing its *Pseudomonas* quinolone signal (PQS), pyocyanin, rhamnolipid, and pyoverdine production (1). In addition, indole has been shown to increase the competitiveness of commensal E. coli with P. aeruginosa by inhibiting its quorum sensing (9). These references are also missing.

Kumar and Sperandio concluded that manipulation of indole concentrations in the gastrointestinal tract by pre- or probiotics that produce indole can limit the virulence of enteric pathogens (4); however, the use of indole as an antivirulence compound was suggested before by our group (1, 10), and indole was used successfully to reduce the virulence of P. aeruginosa in guinea pigs (1). Hence, Kumar and Sperandio are not the first to show that indole reduces EHEC pathogenicity, not the first to indicate that indole is sensed via CpxAR, and not the first to show the importance of indole with non-E. coli strains (both pathogens and nonpathogens).

Lastly, it is worth noting that since indole reduces persistence (11, 12), it may be used by commensal E. coli to kill its dormant competitors (13). In a less belligerent role, indole is also used to keep pathogens asleep without affecting their growth (as it does for P. aeruginosa) to give commensal E. coli an advantage as it first wakes and forages for food (14).
